# Effects of Surgical Correction for the Treatment of Adult Acquired Flatfoot Deformity: A Computational Investigation

**DOI:** 10.1002/jor.21379

**Published:** 2011-02-11

**Authors:** Joseph M Iaquinto, Jennifer S Wayne

**Affiliations:** Orthopaedic Research Laboratory, Departments of Biomedical Engineering and Orthopaedic Surgery, Virginia Commonwealth UniversityRichmond, Virginia

**Keywords:** foot and ankle, pes planus, lateral column lengthening, joint analysis, plantar pressure

## Abstract

Computational models of the foot/ankle complex were developed to predict the biomechanical consequences of surgical procedures that correct for Stage II adult acquired flatfoot deformity. Cadaveric leg and foot bony anatomy was captured by CT imaging in neutral flexion and imported to the modeling software. Ligaments were approximated as tension only springs attached at insertion sites. Muscle contraction of the gastrocnemius/soleus complex was simulated through force vectors and desired external loads applied to the model. Ligament stiffnesses were modified to reflect Stage II flatfoot damage, followed by integration of corrective osteotomies—medializing calcaneal osteotomy (MCO) and Evans and calcaneocuboid distraction arthrodesis (CCDA)—to treat flatfoot. Joint angles, tissue strains, calcaneocuboid contact force, and plantar loads were analyzed. The flatfoot simulation demonstrated clinical signs of disease evidenced by degradation of joint alignment. Repair states corrected these joint misalignments with MCO having greatest impact in the hindfoot, and Evans/CCDA having greatest effect in the mid- and forefoot. The lateral procedures unevenly strained plantar structures, while offloading the medial forefoot, and increased loading on the lateral forefoot, which was amplified by combining with MCO. The Evans procedure raised calcaneocuboid joint contact force to twice intact levels. Computational results are in agreement with clinical and experimental findings. The model demonstrated potential precursors to such complications as lateral tightness and arthritic development and may thus be useful as a predictor of surgical outcomes. © 2011 Orthopaedic Research Society Published by Wiley Periodicals, Inc. J Orthop Res 29: 1047–1054, 2011

Adult Acquired Flatfoot Deformity (*pes planus*) is a multi-stage degenerative disease that leads to improper joint alignment causing pain and affecting foot and ankle mobility. The conditions leading to the onset of flatfoot deformity are not fully understood; however, dysfunction of the posterior tibial tendon is considered a key factor.^1^–^9^ Stage II is characterized by chronic weakness of the tendon coupled with early development of forefoot abduction, midfoot collapse, and hindfoot valgus.^1^,^3^,^6^,^10^,^11^ The morphologic changes occur in conjunction with a weakening of the talonavicular capsule, spring ligament, long and short plantar ligament, and plantar fascia, which degrade as their loading increases during gait and stance in the absence of the functional posterior tibial tendon.^12^,^13^

Several surgical procedures address the weakened posterior tibial tendon and the mal-aligned joint morphology in Stage II. Posterior tibial tendon function is restored by tendon transfer, commonly the flexor digitorum longus.^1^,^3^,^4^,^14^–^16^ Joint alignment is restored with several osteotomies, which exhibit specific strengths and some similar correction of alignment. The medializing calcaneal osteotomy (MCO) is often performed as a hindfoot valgus corrective procedure.^1^,^4^,^5^,^15^,^17^ Additional surgical corrections, classified as lateral column lengthening procedures, counter medial arch collapse, and forefoot abduction by means of lengthening/rotating the mid- and forefoot and are commonly performed in conjunction with an MCO. The Evans opening wedge osteotomy (Evans), and calcaneocuboid distraction arthrodesis (CCDA) are the most common lateral column procedures.^1^,^4^,^5^,^14^,^15^,^17^–^22^ While a wide range of angle changes are reported, correction of excessive forefoot abduction is the most significant outcome for a lateral column procedure.

Complications associated with the Evans and CCDA include non- or delayed union, incision site problems, arthritic development (notably at the calcaneocuboid joint for Evans), and tightness or pain in the lateral foot.^14^,^18^,^23^–^26^ The corrections can alter not only tissue loading and joint contact, but gait and foot biomechanics by impacting plantar pressure distributions.^19^,^24^,^27^–^29^ With flatfoot corrective procedures exhibiting beneficial and detrimental effects, ongoing interest exists to study their biomechanical differences. Our computational models were used previously to demonstrate load sharing and arch stabilizing roles of plantar ligaments.^30^ Joint function is governed by 3D bony anatomy derived from sub-millimeter resolution CT scans, soft tissue behavior, muscle loading, and applied perturbations. In this study, these models were implemented to compare differences in biomechanics of the foot/ankle complex between the normal state and Stage II flatfoot deformity and to assess the effectiveness of common surgical corrective procedures. This was accomplished by measuring radiographic joint angles, ligament strains, joint contact force, and plantar force distribution in the forefoot.

## METHODS

### Model Creation

Following previous methods,^30^ a cadaveric leg was CT scanned using a SOMATOM Sensation 64 Helical Scanner (Siemens, Munich, Germany) to obtain bony anatomy. The scan axis was aligned with the long axis of the tibia to yield isometric voxels (0.6 × 0.6 × 0.6 mm^3^). Processed anatomy was imported to SolidWorks (SolidWorks Corp., Concord, MA) and reassembled in neutral flexion. To incorporate soft tissue contributions, origins and insertions of 144 ligament and tendon bands were identified from anatomic text^31^,^32^ and dissection. These structures were defined as tension only soft tissue arrays in the kinematic simulator COSMOSMotion, a SolidWorks add-on. The 3D complexity of ligaments, particularly for the plantar fascia, plantar ligament, and spring ligament, was captured using multiple elements to describe the major bands of these structures. The wrapping of plantar fascia and long plantar ligamentsaround bone was accomplished with 3D bead elements acting similarly to pulleys.

**Figure 1:**
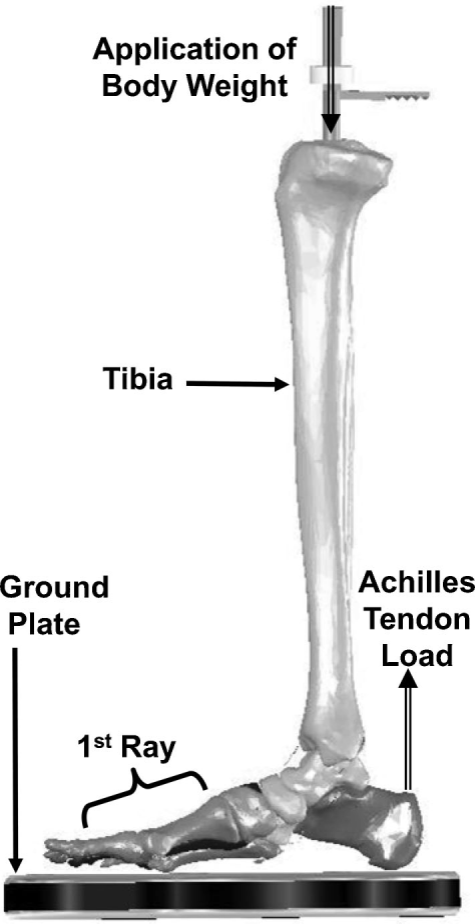
Medial view of foot model. Bony anatomy created from high resolution scans with scan (neutral) orientation preserved through to the 3D model. Modeled bony constraints include proximal load application, ground plate, Achilles tendon action, and tension only ligament arrays (not shown).

Soft tissue stiffnesses^33^ and in situ strains^34^–^36^ were obtained for major structures of the ankle and foot. For structures with no reported parameters in the literature, averages of known structures were instituted.^30^,^37^ To simulate mid-stance conditions, a downward force of 690 N was applied to the proximal tibia, representing an average weight person as used in other studies.^15^,^28^ Achilles tendon load at ½ body weight (345 N) was applied (Fig. 1). During standing and mid-stance, muscle activation beyond that of the gastrocnemius/soleus complex is minimal and was excluded.^12^,^38^ To maintain vertical tibial alignment for stance, the proximal tibia was restrained to one degree of freedom (superior–inferior translation); the remainder of the tibia and fibula interactions were governed by anatomy and associated ligaments.

### Modeling Stage II Flatfoot

Previously reported MR damage^13^ to key structures was used to guide the simulation of ligament degeneration with Stage II flatfoot deformity. The extent of altered appearance of ligament tissue and full thickness tearing was categorized into stages of overall damage, which served as a template to adjust stiffness values of affected ligaments (Table 1). To distinguish among models, “normal” refers to intact anatomy with normal ligament properties, “flatfoot” refers to intact anatomy with reduced stiffness ligaments. “MCO,” “Evans,” “CCDA,” etc. refer to the flatfoot model with specific bony procedure(s).

**Table 1:** Soft Tissue Structures Affected by Flatfoot Deformity, with Corresponding Damage Levels,^13^ Selected Stiffness Reduction Amounts, and Final Flatfoot Stiffnesses

Structure	Damage Scale	Stiffness Adjustment	Flatfoot Stiffness (N/mm)
Superomedial spring	Stage IV	−7/8th	39
Inferomedial spring	Stage II	−3/8th	94
Talocalcaneal interosseus	Stage I	−1/8th	236
Plantar fascia	Stage I	−1/8th	175
Plantar metatarsocuneiform	Stage 0	None	90
Plantar naviculocuneiform	Stage 0	None	180
Long and short plantar	Stage 0	None	240
Deep deltoid	Stage 0	None	200
Anterior superficial deltoid	Stage I	−1/8th	70
Posterior superficial deltoid	Stage 0	None	117

Structures represent distinct portions of ligaments as seen with deltoid and spring ligament portions where damage was noted in only some of the structure.

### Modeling the MCO

**Figure 2:**
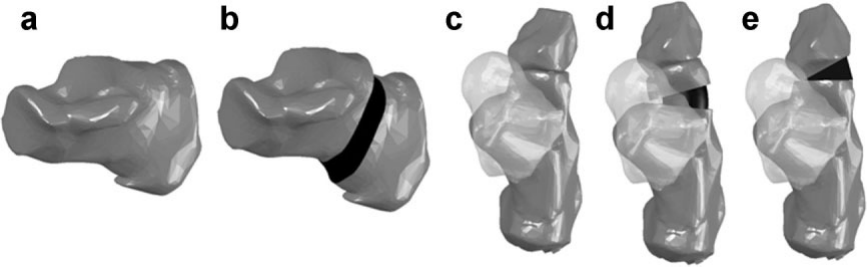
Surgical procedures performed on the foot to correct flatfoot deformity: (a) Intact calcaneus from an oblique anteromedial view; (b) calcaneus with 1 cm MCO, shown with cut face darkened; (c) intact hindfoot with cuboid from a superior view; (d) Evans modified hindfoot with darkened 1 cm wide wedge inserted between body and calcaneal fragment (note cuboid rotation); (e) CCDA modified hindfoot with darkened 1 cm wide wedge inserted between shaved cuboid and calcaneus. The talus is made semi-transparent for clarity purposes. Note smooth appearance of Evans wedge due to trimming to prevent projection into the subtalar joint.

This osteotomy was created by directly modifying the calcaneus. A plane was positioned to isolate the Achilles tuberosity from the body of the calcaneus as is performed clinically. The fragment was moved medially 10 mm and reaffixed to form the MCO (Fig. 2a,b).^22^,^28^,^34^,^39^,^40^

### Modeling the Evans Procedure

The anterior facet of the calcaneus was detached ∼10 mm behind the articular surface. The fragment was then rotated internally about its medial border until a 10 mm wide, full depth (25 mm) triangular wedge could be inserted between the fragment and body of the calcaneus.^11^,^19^,^27^,^28^,^38^,^41^ Wedge, fragment, and body were then fused to form the Evans osteotomy (Fig. 2c,d).

### Modeling the CCDA

Approximately 3 mm of the most superficial shared articular joint surfaces of the cuboid and calcaneus were removed to create flat planes. The cuboid was then rotated internally about its medial border with the calcaneus such that a 10 mm wide, full depth (25 mm) triangular wedge (with 4 mm removed to eliminate protrusion into the subtalar joint) could be placed between the two bones.^14^,^19^,^29^,^41^ These were then fused to form the CCDA (Fig. 2c,e).

### Simulations and Measurements

**Figure 3:**
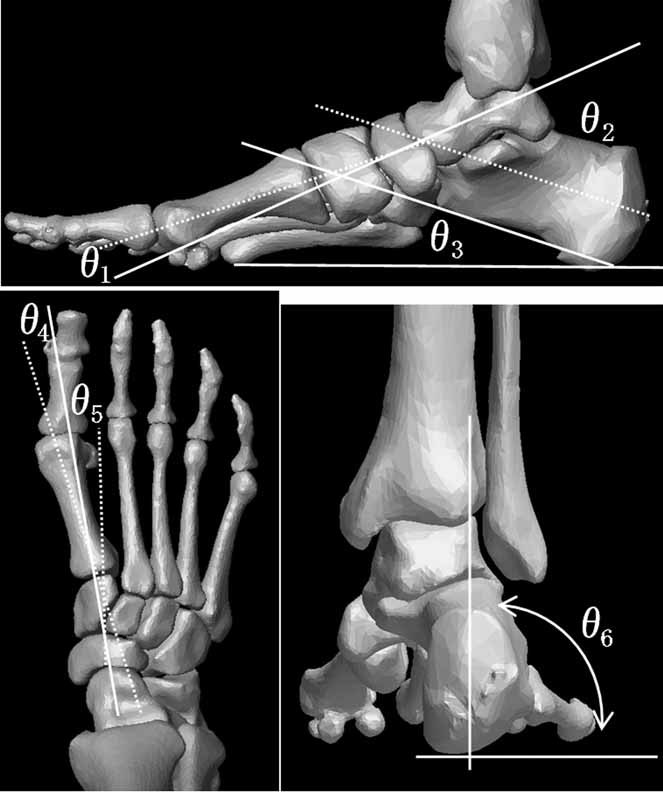
Radiographic views and associated joint angles. Top: Lateral view of the foot showing Talo-1st MetaTarsal (L-T1MT) angle, *θ*_1_; TaloCalcaneal (L-TC) angle, *θ*_2_; and Calcaneal Pitch (L-CP) angle, *θ*_3_. Bottom left: 70° raised AP view of the midfoot showing Talo-1st MetaTarsal (AP-T1MT), *θ*_4_ and TaloNavicular angle (AP-TN), *θ*_5_. Bottom right: posterior view of the calcaneus showing hindfoot varus/valgus measured from the lateral aspect, *θ*_6_.

Seven configurations were simulated: normal, flatfoot, and flatfoot with various osteotomies (MCO, Evans, CCDA, Evans and MCO, CCDA and MCO). Radiographic views were created and referencing markers added for measures used in flatfoot diagnosis.^1^–^3^,^6^,^10^–^12^,^14^,^15^,^23^,^41^ Such markers described the Talo-1st MetaTarsal (L-T1MT), Calcaneal Pitch (L-CP), and TaloCalcaneal (L-TC) joint angles in the lateral view (Fig. 3a) and the Talo-1st MetaTarsal (AP-T1MT) and TaloNavicular coverage (AP-TN) angles in the AnteroPosterior (AP) view (Fig. 3b). Calcaneal varus/valgus was measured from a posterior view (Fig. 3c). Soft tissue strain was calculated from elongation of ligament arrays relative to their resting state with no axial loading. These strains were analyzed across the width of the long plantar ligament and plantar fascia, as strains in different bands of these structures have been studied in literature. Resultant contact force can be determined for any joint within the foot/ankle complex. However, focus was placed on the forces generated at the calcaneocuboid articulation due to a susceptibility for arthritis following Evans procedures.^18^,^26^,^41^,^44^ Plantar loads were measured through bony ground contact at the distal rays and calcaneus.

## RESULTS

### Radiographic Joint Angles

**Figure 4:**
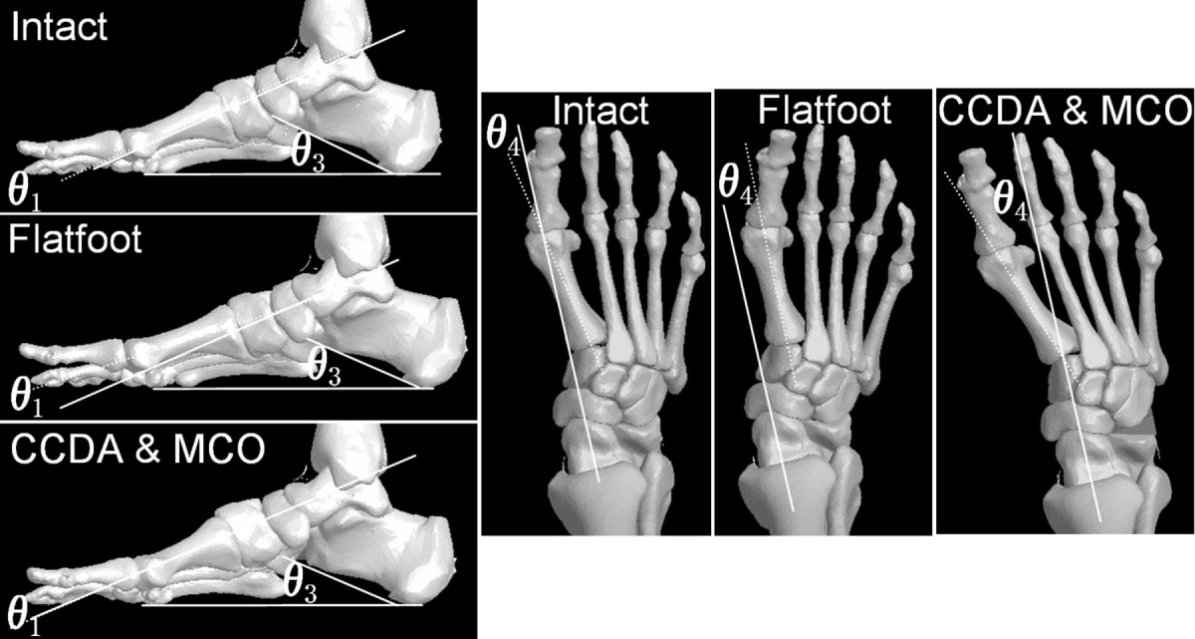
Radiographic views of the intact, flatfoot, and flatfoot treated with CCDA and MCO to highlight some of the angular changes resulting from surgical correction. Lateral views show representations of L-T1MT angle, *θ*_1_ and L-CP, *θ*_3_ (left series); AP views include a representation of the AP-T1MT angle, *θ*_4_ (right series). Note large changes in AP forefoot ab/adduction and subtle changes laterally in medial column alignment and height.

The normal foot exhibited joint angle measures comparable to those seen clinically with the exception of L-TC and L-CP, which were slightly less than reported averages (Table 2). Simulated flatfoot showed all of the trends seen clinically: forefoot abduction (decreasing AP-T1MT and AP-TN angles), midfoot collapse (decreasing L-CP and L-T1MT angles), and hindfoot valgus (increasing Hindfoot angle) (Fig. 4).

**Table 2:** Joint Angles (Fig. 2), Measured for Normal and Flatfoot Surgical Stages in Comparison to Values in Clinical Literature (*Italicized*)

		Flatfoot					
		
Joint Angle (°)^a^	Normal, Intact	Intact	MCO	Evans	CCDA	Evans and MCO	CCDA and MCO
L-T1MT (*θ*_1_)	0.5	−8.6	−3.7	2.9	6.8	1.2	0.8
	*−3.3 ± 4.9*^10^	*−17.5 ± 6.4*^10^		*−7.7*^42^	*−3.9 ± 3.2*^14^		
	*0.0 ± 0.5*^41^	*−5.6 ± 1*^41^		*−8.4*^11^			
		*−14.2 ± 7.1*^14^					
		*−26.6*^42^					
		*−19.7*^11^					

L-TC (*θ*_2_)^b^	39.4	41.0	37.9	43.2	37.5	41.3	35.8
	*50.3 ± 5.6*^10^	*36.2 ± 30.5*^10^					
	*45.8 ± 0.4*^41^	*44.7 ± 0.7*^41^					

L-CP (*θ*_3_)	16.6	14.0	13.1	19.5	15.7	17.0	13.7
	*22.8 ± 4.7*^10^	*16.3 ± 6.3*^10^		*14*^11^			
		*3.2*^11^					

AP-T1 MT (*θ*_4_)	7.2	−1.7	7.2	11.9	16.8	13.5	20.5
		*−15.3 ± 8.7*^14^		*−7.7*^42^	*−4.1 ± 3.8*^14^		
		*−27.1*^42^		*−11*^11^			
		*−26.8*^11^					

AP-TN (*θ*_5_)	−7.0	−8.9	−6.5	2.4	−2.4	3.5	−0.4
	*−10.4 ± 4.2*^10^	*−22.3 ± 6.7*^10^					

Hindfoot (*θ*_6_)^b^	93.4	96.4	87.7	94.7	93.1	90.9	86.9
	*95* (*93–97*)^c^^10^	*99* (*94–105*)^c^^10^					

Angles are: Lateral Talo-1st MetaTarsal (L-T1MT), *θ*_1_; Lateral TaloCalcaneal (L-TC), *θ*_2_; Lateral Calcaneal Pitch (L-CP), *θ*_3_; AnteroPosterior Talo-1st MetaTarsal (AP-T1MT), *θ*_4_; AnteroPosterior TaloNavicular (AP-TN), *θ*_5_, Hindfoot varus/valgus (Hindfoot), *θ*_6_.

a Negative values denote crossing a neutral axis: for L-T1MT, this signifies a drooping medial arch; for AP-T1MT and AP-TN, this signifies abduction.

b Neither the L-TC nor hindfoot angles have an associated neutral axis. L-TC values greater than normal indicate talar plantarflexion. Hindfoot less or greater than intact indicate more varus and valgus, respectively.

c 90° was added to hindfoot angles to transform them to the coordinate system used in simulation.

With simulation of the MCO, improvement toward normal was seen in some angles (L-T1MT, AP-T1MT, and AP-TN) with overcorrection or a further decrease in others (L-TC and L-CP, respectively). Overcorrection of calcaneal alignment into varus was noted (Hindfoot angle less than 90°). Lateral column lengthening procedures exhibited correction beyond normal levels at several joint angles (L-T1MT, AP-T1MT, and AP-TN) with mixed corrections in others (L-TC and L-CP). Calcaneal alignment (Hindfoot) was corrected toward normal levels.

For combined procedures, medial column alignment (L-T1MT) was corrected to near normal levels compared to the MCO or lateral procedures alone. For the other lateral measures, improvement toward normal was seen for L-CP, but L-TC did not improve from the flatfoot case with the Evans and MCO procedure. CCDA and MCO each overcorrected L-TC alone such that their combination further exacerbated this overcorrection, while L-CP did not improve from the flatfoot case and worsened with CCDA. For either lateral column procedure, the addition of the MCO further increased forefoot correction into adduction (larger AP-T1MT and AP-TN angles). The overcorrection of calcaneal alignment (Hindfoot) into varus with the MCO was mitigated by combining with the Evans procedure, but not with CCDA combination procedure.

### Ligament Strain

Medial/lateral tissue strain was uniform to within ±2% strain in the normal simulation (Table 3). With flatfoot, strain in the medial band of the plantar ligament increased 180% over normal levels; an 80% increase occurred in the plantar fascia. Intermediate bands were affected less than medial, and lateral bands were affected the least. The model exhibited elongation of lateral bands of the long plantar ligament and plantar fascia and slackening of medial bands, with the addition of lateral column lengthening procedures. From MCO, to lateral column procedures, to combination procedures, the increased strain in the medial band of the long plantar ligament in flatfoot steadily declined to 40% above normal levels with CCDA and MCO. The medial band of the plantar fascia dropped to 87% of normal with MCO, and continued to drop to 10% of normal levels with CCDA and MCO. For the long plantar ligament, the lowest lateral strain was with lateral column procedures alone (+130%), and the largest was with CCDA and MCO (+250%). For the plantar fascia, the lowest lateral strain was seen with MCO (+30%); all other procedures demonstrated a similar increase (+47%).

**Table 3:** Soft Tissue Strains Calculated From Resting to Loaded, in % Strain, for the Long Plantar Ligament and Plantar Fascia for All Simulations

		Flatfoot					
		
% Strain in Ligament Structures	Normal, Intact	Intact	MCO	Evans	CCDA	Evans and MCO	CCDA and MCO
Long plantar array							
Long plantar 1 (med)	1.0	2.8	2.1	2.4	2.1	1.9	1.4
Long plantar 2	1.0	2.8	2.5	2.7	2.3	2.2	2.2
Long plantar 3	1.5	2.8	2.5	3.0	2.5	2.7	2.0
Long plantar 4	1.8	2.3	3.1	3.4	2.9	3.3	2.2
Long plantar 5	1.7	2.9	2.7	3.5	2.9	3.5	2.6
Long plantar 6	1.8	2.7	3.0	3.9	3.2	4.0	3.2
Long plantar 7	0.6	1.2	2.1	2.7	2.0	3.5	3.1
Long plantar 8 (lat)	1.7	1.7	5.3	4.1	4.0	5.6	6.0
Plantar fascia array							
Plantar fascia 1 (med)	3.9	7.2	3.4	2.5	2.0	1.6	0.4
Plantar fascia 2	2.1	4.3	2.1	2.2	1.8	1.2	0.5
Plantar fascia 3	2.7	4.5	3.5	3.5	3.3	2.5	1.5
Plantar fascia 4	2.7	4.0	3.6	4.7	4.1	5.1	3.8
Plantar fascia 5 (lat)	3.2	3.7	4.2	4.7	4.7	4.7	4.8

Ligament elements are listed medial (med) to lateral (lat).

### Calcaneocuboid Contact Load

Joint contact load between the calcaneus and cuboid increased 16% from normal to flatfoot. The load returned to near normal levels (increase of 1%) with MCO. The Evans procedure alone increased the calcaneocuboid joint load by 111% over normal levels while this increase was 93% for Evans and MCO.

### Plantar Ground Loads

In the model, flatfoot shifted loads toward the medial forefoot, with a slight increase in 1st ray contact load and doubling of 2nd ray load (Table 4). The corrective osteotomies shifted loads from the medial forefoot to the lateral forefoot, with greater impact for combination lateral column lengthening and MCO procedures. The MCO reduced the increase in medial forefoot loading caused by flatfoot to levels half of normal while also doubling lateral forefoot loading. For the Evans and CCDA procedures, this medial to lateral redistribution of load was much more pronounced. The combination procedures redistributed the plantar ground loads the most. The medial forefoot was nearly unloaded with these procedures. The lateral forefoot load was also more than doubled compared to both normal and flatfoot. The 5th ray always exhibited greater ground contact load than the other rays.

**Table 4:** Contact Loads (N) between the Ground and Plantar Aspect of the Foot

		Flatfoot					
		
Load in Planter Region	Normal, Intact	Intact	MCO	Evans	CCDA	Evans and MCO	CCDA and MCO
Ray 1	116	125	58	38	18	8	0
Ray 2	8	16	2	6	9	4	0
Ray 3	40	36	23	38	47	25	26
Ray 4	24	30	79	52	49	56	53
Ray 5	61	60	117	122	133	160	177
Calcaneus	428	422	410	431	448	443	441

Listed are loads under rays 1–5 and the heel.

## DISCUSSION

### Radiographic Joint Angles

With few exceptions, the normal and flatfoot simulations agreed well with the literature. The 9.1° decrease in L-T1MT angle in the flatfoot model corresponded with the decrease documented in the literature (5.6°–14.2°).^10^,^41^,^42^ The L-CP decreased by 2.6° (16.6°–14°) falling within the reported standard deviations for this angle (6.5° drop with ∼5° std. dev.).^10^ The L-TC angle increased in the model by 1.6° with flatfoot while the reported trend is a decrease (14.1° drop from 50.3 to 36.2 and a 1.1° drop from 45.8 to 44.7). However, the standard deviations for the greatest drop are large (±30.5).^10^,^41^ Additionally, the sensitivity of L-TC in identifying deformity and correction has been questioned.^11^ The AP angles shifted into greater abduction in the simulated flatfoot as in other studies, although AP-T1MT was less than reported for flatfoot. The shift for AP-TN was also less than reported (−1.9° vs. −11.9 with ∼5° std. dev.).^10^,^14^,^42^ A smaller foot abduction in the flatfoot simulation could be due to an under representation of weakened ligaments or from assumed soft tissue properties, which created a slight adduction in the intact foot thus lessening abduction in flatfoot. Hindfoot angles were within reported ranges (93.4° model vs. 95° reported and 96.4° model vs. 99° reported for intact and flatfoot, respectively).^10^

The MCO improved all joint angles in the model and the literature except for the L-CP. This angle is influenced by calcaneal varus rotation, which moves the anatomical landmarks inferiorly. In flatfoot deformity, the line of action for the Achilles tendon is laterally located, which provides positive feedback to worsen hindfoot valgus. Clinically, the MCO is used to correct Achilles tendon pull by restoring neutral alignment to the muscle line of action^5^,^27^,^39^,^40^ although quantitative measures are few in the literature. Simulation of the MCO brought the calcaneus into 2.3° of varus, overcorrecting from normal (5.7° varus movement from 93.4° to 87.7) and from flatfoot (8.7° varus movement from 96.4° to 87.7°).

In the model and literature, both the Evans and CCDA improve L-T1MT angle from flatfoot. For Evans, this improvement was 11.5° in the model compared to a range of 11.3–18.9° in the literature.^11^,^14^,^18^,^23^,^38^,^42^ For CCDA, the improvement was 15.4° in the model versus 10.3° in the literature (std. dev. of ∼5°).^14^ The final angle of both Evans and CCDA simulations ended in slight overcorrection from normal, however, large variation in L-T1MT exists for flatfoot in the literature. L-TC correction with simulated lateral column procedures is difficult to assess due to large standard deviations in the literature.^18^,^38^ Simulated improvement of L-CP is also noted with either lateral column procedure.^18^,^38^ The AP angles experienced the greatest correction from lateral column lengthening.^11^,^14^,^18^,^23^,^38^,^42^ The AP-T1MT angle improved with Evans, 13.6° compared to reported improvement of 15.8 to 19.4° (no std. dev. available). CCDA improved this angle 18.5° in simulation and 11.2° (∼6° std. dev.) in the literature. Combination procedures are used clinically, with the MCO treating hindfoot valgus and lateral column procedure correcting forefoot abduction,^1^,^3^,^5^,^23^ but reported angular corrections for specific combinations are not widespread.

### Ligament Strain

The medial band of the plantar fascia exhibits slackening with MCO and CCDA but strain has not been reported.^22^ Such medial slackening is also reported for the long plantar ligament with Evans procedure^43^ and compares well qualitatively with the medial decrease in strain found in all simulations for these structures with the osteotomies. Also, the increase in lateral strain is reflected in the literature where the Evans procedure increased the elongation of the lateral portion of long plantar ligament.^43^ These findings may correlate to reported lateral foot pain following surgery.^23^

### Calcaneocuboid Contact Load

Lateral column procedures can potentially cause accelerated arthritic development in the mid- and hindfoot, particularly with the Evans osteotomy.^25^,^26^ The calcaneocuboid joint is of particular interest because arthritic development in this joint is noted at follow-up with patients who received the Evans procedure.^18^,^26^,^42^ Experimental findings showed a quadrupling of contact load at this joint, and increases in contact area and total and peak pressures, but with large variability.^25^ Joint contact force for Evans and Evans and MCO in the model more than doubled compared to levels at normal, flatfoot, or MCO alone, a level that is within the experimental standard deviation.^25^ Such increases may well be correlated with future joint degeneration.

### Plantar Ground Loads

With an MCO, a drop in percent bodyweight carried by the 1st metatarsal and an increase to the 4th and 5th metatarsals was seen in a cadaver experiment.^19^ Additional experimentation found a significant decrease of average pressure *and* a reduction of contact area to the 1st and 2nd metatarsal heads with an MCO, and an increase in lateral forefoot peak pressure with no reported change in lateral contact area.^27^ This correlates well with the simulated MCO where the 1st metatarsal load decreased by 50%, and 5th metatarsal load increased by 50%.

With respect to lateral column procedures, average pressure decreased in the cadaveric 1st metatarsal by 33 ± 30% for Evans procedure and 21 ± 41% for CCDA while average pressure increased in the 5th metatarsal by 46 ± 42% (range −4% to 141%) for the Evans procedure and 104 ± 58% (range 9–216%) for the CCDA.^28^ This correlates well with the model where the contact loads decreased under the 1st metatarsal heads by 67% for Evans and 84% for CCDA, while the contact loads increased by 100% under the 5th metatarsal for Evans and 122% for CCDA. The experimental pressure measurements were made using a fixed area under the metatarsal heads, although it is not known whether the same number of sensels remain loaded.^28^

For combination procedures, an increase in lateral forefoot pressures was seen in the cadaver after both Evans and CCDA with and without MCO, with no significant difference between procedures and no reported change in contact area.^29^ In the model, 5th metatarsal contact force was greatest with either Evans (160% increase) or CCDA (190% increase), both with MCO. For the same cadaveric study, a significant decrease in medial forefoot pressures was found for CCDA, Evans, and CCDA and MCO with a corresponding drop in contact area for CCDA and Evans, both with MCO.^29^ In the model, 1st metatarsal contact force was reduced with Evans (93% decrease) and CCDA (100% decrease), both with MCO.

The model includes certain assumptions. The model represents one leg, which is assumed normal and comparable to separate studies in the literature. While boundary conditions were selected to approximate in situ conditions, loading conditions in the literature varied, including muscle activation, which may cause discrepancy in compared results. Flatfoot ligament attenuation based on qualitative MR appearance can only be approximated from live subjects. Ligament in situ strains and stiffness are not reported for most structures within the foot/ankle complex and were thus assigned as averages of reported values. This was considered a reasonable assumption given the large variations in stiffness values in the literature^33^–^36^ but could lead to differences in compared results. Linear behavior was assumed for these structures, which could overestimate their restraining role. Our prior work^30^ however showed that variations of ligament stiffness values by ±43% do not change conclusions from the model. While most foot and ankle ligaments are short and do not appreciably wrap around bone during function, others do exhibit wrapping. These large ligaments (plantar fascia and long plantar ligament) were modeled with small bead elements to allow wrapping.^37^ Also, the articular cartilage was not captured by CT imaging, which could change joint surface anatomy. However, cartilage thickness variations are typically <0.6 mm.^45^,^46^ Finally, in the absence of plantar tissues (e.g., skin, fascia, fat pads), contact loads but not pressures are calculated between the bones and ground platform. These loads cannot be directly compared to experimentally measured contact pressures without knowing contact areas. An indirect comparison may be useful for investigating changes in contact parameters between states. For example, when combined with kinematics, the calcaneus rolling toward varus (*θ*_6_ decreasing from 96.4° in flatfoot to 87.7° with MCO) would likely load the more lateral regions of the heel pad. This would compare favorably with the lateral shift in pressure seen experimentally.^27^

This computational model is an aid in understanding the complex interaction between corrective technique and potential precursors (e.g., increased joint loads and soft tissue strains) to future complications such as arthritis and pain. The interplay among joint angles, ligament strain, joint contact, and ground contact changes is still largely unknown and difficult to unravel clinically. These measurements illustrate the capability of this parametric model to capture the joint degradation effects associated with flatfoot, along with the multi-factorial biomechanical impact of controversial treatments for flatfoot deformity. The degree of deformity in the model suggests that standard sizes for the MCO and lateral column procedures (or possibly using them together) could lead to overcorrection of deformity. Indeed, such issues of wedge size and technique are explored in the literature when addressing each presenting flatfoot.^1^,^3^,^18^,^23^,^26^,^40^ Future development of this model with subject specific anatomies used with clinical observation could aid in understanding the potential complications of these procedures and in the discovery of new treatments and tailoring of current ones.

## Abbreviations

MCO: medializing calcaneal osteotomy
CCDA: calcaneocuboid distraction arthrodesis
L-T1MT: MedioLateral Talo-1st MetaTarsal angle
L-TC: MedioLateral TaloCalcaneal angle
L-CP: Mediolateral Calcaneal Pitch
AP-T1MT: AnteroPosterior Talo-1st MetaTarsal angle
AP-TN: AnteroPosterior TaloNavicular angle
